# Granulocytic Sarcoma of the Male Breast in Acute Myeloblastic Leukemia with Concurrent Deletion of 5q and Trisomy 8

**DOI:** 10.1155/2012/194312

**Published:** 2012-07-17

**Authors:** Muhammad Rizwan, Md. Monirul Islam, Zia ur Rehman

**Affiliations:** ^1^Department of Internal Medicine, Vidant Medical Center Greenville, NC 27835, USA; ^2^Division of Pulmonary and Critical Care Medicine, East Carolina University and Vidant Medical Center, Greenville, NC 27835, USA; ^3^Departments of Pulmonary, Critical Care and Sleep Medicine, Brody School of Medicine, East Carolina University and Vidant Medical Center, Greenville, NC 27835, USA

## Abstract

We describe a unique case of Granulocytic Sarcoma (GS) in a male, who presented to us with a painless right breast mass without any prior history of Leukemia. GS is an extramedullary tumor of myeloproliferative precursors and may involve multiple sites of the body, but involvement of male breast is extremely rare. In the absence of clinical history or hematological abnormality, GS may be misdiagnosed, depending on the degree of myeloid differentiation present within the tumor. Often it is misdiagnosed as lymphoma. Diagnosis is made by finding eosinophilic myelocytes, myeloperoxidase, chloroacetate esterase staining, and lysozyme immunostain. Chemotherapy regimens similar to acute myeloid leukemia are recommended to treat GS. Recognition of this rare entity is important because early, aggressive chemotherapy can induce regression of the tumor and improve patient longevity.

## 1. Introduction

Granulocytic sarcoma is a rare extramedullary tumor consisting of primitive myeloid cells. It is also known as chloroma, myeloblastoma, extramedullary leukemia, and myeloid sarcoma. This localized growth of immature myeloid precursors superficially resembles sarcoma. The tumor is a solid collection of immature malignant white blood cells (myoblasts) occurring outside the bone marrow, and is a rare manifestation of acute and chronic leukemias. It can occasionally precede the development of systemic disease by weeks to years. GS was named as chloroma, because of its greenish color. The histology of this unusual tumor was first described by Burns in 1811 [1]. The term “chloroma,” literally implying “green tumor”, was suggested by King in 1853 (Greek *chloros*, meaning green) [2]. The green coloration is now known to be due to the presence and oxidation of the myeloperoxidase enzyme. More than a century later, in 1966, Rappaport coined the currently accepted term, “granulocytic sarcoma,” recognizing the fact that the green color is not universal [3].

These tumors may arise *de novo*, or could be associated with other hematologic disorders such as acute myelogenous leukemia, myelodysplastic syndrome, or with myeloproliferative disorders such as chronic myelogenous leukemia, polycythemia vera, hypereosinophilia, and myeloid metaplasia. GS can develop at any anatomic site and its distribution commonly includes bone, nerve, lymph node, and skin, but may involve a variety of soft tissues. In men, the breast has been reported to be an uncommon site for granulocytic sarcoma [4–6]. This extramedullary neoplasm may represent a diagnostic and therapeutic dilemma for both the hematopathologist and oncologist.

## 2. A Case Report

A 56-year-old Caucasian male presented with right breast enlargement of one month's duration. It started with swelling under the right nipple that had progressed into a large mass. The mass was painless but caused discomfort due to cosmetic reasons. Over the past week the patient had also noticed a change in skin color to orange and then to red, overlying the lower outer quadrant of the right breast. The patient denied any history of trauma, fever, weakness, recent weight loss, or loss of appetite. He complained of mild left hip and upper thigh pains of few days duration. His past medical history was non-contributory. Family history was significant for breast cancer in an older sister at age 55 years.

**Figure 1:**
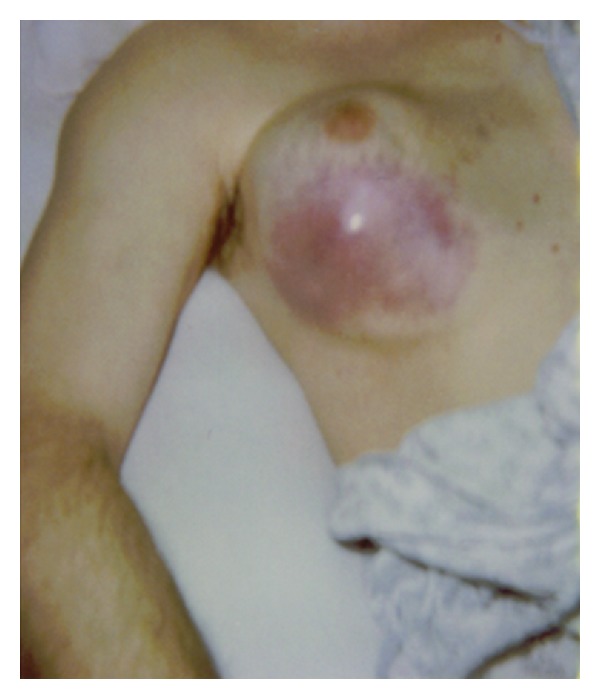
Right breast mass with overlying skin discoloration.

Physical examination revealed a 13 cm × 15 cm, well-circumscribed, nontender mass, hard in consistency, and fixed with the overlying skin and underlying structures (Figure 1). The skin overlying the mass was pinkish. There was no nipple retraction or discharge. The left breast structures were within normal limits. There was mild conjunctival pallor, but no palpable axillary, inguinal, popliteal, or supraclavicular lymph nodes.

**Figure 2:**
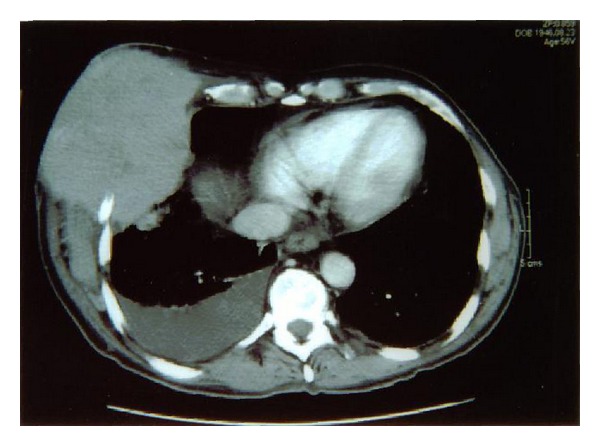
CT chest showing eroding mass lesion.

Laboratory studies showed hemoglobin of 9.6 g/dL hematocrit of 28.7%, platelet count of 102 × k/*μ*L and WBC of 22 × k/*μ*L with differential of 44% neutrophils, 30% bands, 20% lymphocytes, 4% monocytes, and 2% eosinophils. CT scan of the chest revealed a large, 13 cm × 9 cm mass involving the right anterior and lateral chest walls, eroding into the thoracic cavity (Figure 2). The mass demonstrated areas of low density suggestive of necrosis. The fifth and sixth right ribs had been eroded by the mass lesion. An adjacent fluid collection was visible. There was no evidence of lung involvement but a 1.5 cm right adrenal gland nodule was noted. Bone scan performed with gamma camera revealed a suspicious focus of increased uptake in the proximal left femoral shaft and small focus of increased uptake in the superior occipital skull.

**Figure 3:**
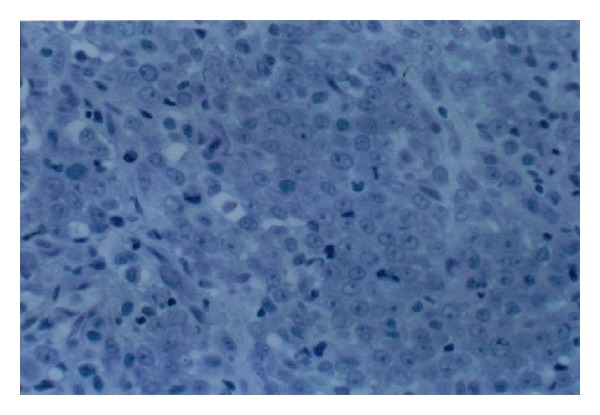
Trucut biopsy of the mass. Moderate-to-large size neoplastic cells with slightly irregular nuclei and conspicuous nucleoli.

**Figure 4:**
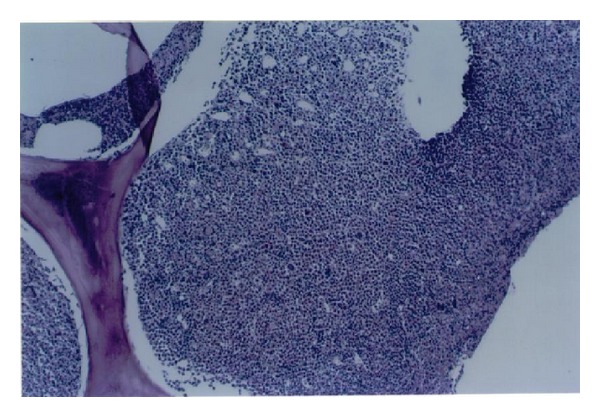
Bone marrow biopsy showing hyperplastic marrow with high M/E ratio.

Trucut biopsy of the right breast mass showed myeloid sarcoma (Figure 3). Bone marrow biopsy showed hyperplastic marrow with high M/E ratio with shift to left (Figure 4). Bone marrow immunohistochemical studies were positive for lysozyme, myeloperoxidase, CD15, CD34, and CD43. These findings were consistent with a Myeloid Neoplasm, but not specific for a certain type. The differential diagnosis proposed was chronic myeloid leukemia (CML), a myelodysplastic/myeloproliferative disease (e.g., chronic myelomonocytic leukemia, or CMML), and less likely, a myelodysplastic syndrome. With pending chromosome analysis, patient was started on therapeutic trial of Gleevec 600 mg orally daily and Allopurinol 300 mg orally daily. Arrangements were made for radiation therapy to the mass to improve local symptoms and reduce the size of the tumor. Patient did well on this therapy except for developing anemia for which he was transfused.

Three days later, the patient sustained a fall resulting in an acute transverse fracture of the proximal left femoral neck. Left hip arthroplasty was performed. Bone reamings from left femur, which were sent for studies, showed diffuse infiltration of neoplastic cells with extensive necrosis. Neoplastic cells were moderate to large in size and had modest to moderate amount of pink cytoplasm and slightly irregular nuclei with fine chromatin and conspicuous nucleoli. Mitotic activities were frequently present, consistent with myeloid neoplasm. The morphologic findings were suggestive of the same neoplastic process as the previous bone marrow biopsy and breast biopsy. Bone marrow chromosome analysis report came back as well and revealed complex clonal aberrations including deletion of 5q, trisomy 8, and additional material on the short arm of chromosome 17.

It was planned to start patient on AML-type chemotherapy upon his recovery, but unfortunately, postoperatively the patient developed respiratory failure and was transferred to intensive care unit on assist control ventilation. He was empirically started on Primaxin and Vancomycin for the possibility of healthcare associated pneumonia. Lovenox dose was changed from prophylactic to therapeutic for possible pulmonary embolism. CT angiogram of chest was obtained which showed bilateral intraluminal filling defects in second order branches consistent with pulmonary emboli. Multiple attempts to wean him off the ventilator were unsuccessful. Patient expired five days after surgery.

## 3. Discussion

Isolated granulocytic sarcomas are rare tumors associated with myeloproliferative disorders or leukemias. They have been reported both before and after the hematological diagnosis [4]. Usually, there is known bone marrow involvement at the time of presentation and a clinical diagnosis is made. Thus, presentation can occur either prior to or in association with the underlying myeloid disorder or upon relapse.

This group of disorders can involve myeloblasts and neutrophil precursors, monoblasts, or can be a trilineage myeloid tumor of erythroid precursors, megakaryocytic precursors, and granulocytic precursors [5]. It is frequently identified in patients with chronic myeloid leukemia, myeloproliferative disorders, or myelodysplasia, but most commonly these tumors represent relapse or the initial presentation of acute myeloid leukemia. Incidence is increased in FAB M4/M5 types, CD56 (+) blasts, cytogenetic abnormalities: t (8 : 21), inversion 16, infant leukemia, 11q abnormalities, cellular immune dysfunction, and with new treatments like allogeneic stem cell transplantation [5]. It has also been reported as an isolated mass without prior history or subsequent development of leukemia [5, 7].

Overall, GS has been classified into four categories: (a) primary GS, (b) GS as a complication of acute myeloblastic leukemia (AML), (c) GS as isolated recurrence of AML particularly during bone marrow remission and not followed by medullary relapse, and (d) GS with concurrent bone marrow relapse of AML [8]. Our patient belongs to category b, GS as a complication of AML as per clinical history and lab results.

GS is more common in males. Median age for males and females is 32 and 34, respectively, and most of the patients are in the age range of 20–44 [9]. GS can present as a single lesion or multiple lesions. Sizes can vary greatly and some are large enough to cause compression symptoms or signs according to their localization. GS is widespread and can involve almost any part of the human body. Common sites of involvement are lymph nodes 15%, skin 14%, head, and spinal cord 13%, small intestine 11%, mediastinum 10%, bone 9%, ovary and uterus 9%, and others 19% [9].

Breast involvement by GS is rare and that of the male is extremely rare. Gynecomastia may be the first manifestation of this disease or it may present as an isolated breast mass [6, 10]. In the absence of clinical history or hematological abnormality, granulocytic sarcoma may be misdiagnosed, depending on the degree of myeloid differentiation present within the tumor. A careful evaluation of the breast mass is important, and GS should be differentiated from other nonmammary malignancies of the breast; primary and secondary breast lymphoma, primary axillary nodal lymphoma, metastatic acute lymphatic leukemia, metastatic plasmacytoma, primary angiosarcoma, metastatic rhabdomyosarcoma, hematogenous metastasis from primary lung, ovarian, cervical, thyroid and colonic carcinoma, malignant melanoma, carcinoma of the nasal cavity, and adenocarcinoma of unknown primary [11].

### 3.1. Diagnosis

Mammographically, breast GS are noncalcified irregular masses with poorly defined feathery margins [11]. MRI will only show a mass lesion but is helpful in evaluating response to treatment and detect the nonpalpable relapse of the tumor [12].

GS infiltrates in tracts and tissue planes, usually preserving the tissue architecture without extensive tissue destruction and tumor necrosis. Histopathologically, these tumors have been classified into well differentiated, poorly differentiated, and blastic types depending upon the level of myeloid maturation by hematoxylin-eosin and PAS stained sections [6, 13]. Histologic diagnosis remains difficult. The presence of eosinophilic myelocytes has traditionally been one of the most reliable histologic findings in making a diagnosis of GS. Tumors are considered well differentiated if numerous eosinophilic myelocytes can be seen in any section of a given case. Diagnosis can be confirmed with myeloperoxidase (MPO), chloroacetate esterase staining (leder stain), and lysozyme immunostain(Ly). Histochemical staining to demonstrate the presence of MPO is the most sensitive and specific antibody test for detection of myeloid differentiation [14]. Electron microscopy, which reveals azurophilic granules consistent with promyelocytes, helps confirm diagnosis [7] but is rarely performed due to cost and availability. Specific markers of cluster differentiation (CD) typical of myeloid lineage can also be demonstrated. For example, Chen and colleagues found that MPO was positive in 97% of cases, Ly in 93%, CD34 in 47%, CD45 in 84%, CD43 in 97%, CD68 in 93%, Bcl-2 in 68%, Tdt in 37%, CD79a in 20%, CD20 in 10%, CD3 in 10%, and c-kit (CD117) reactivity in 87% of the cases [15].

### 3.2. Differential Diagnosis

Differential diagnosis of GS on fine needle aspiration (FNA) include large cell non-Hodgkin lymphoma, lymphoblastic lymphoma, Hodgkin lymphoma, extramedullary hematopoiesis, poorly differentiated carcinoma, infection, inflammation, plasmacytoma, and malignant melanoma [13, 16].

In differential diagnosis, immunohistochemistry is the most powerful tool and is regarded as essential for diagnosing granulocytic myeloid tumor and distinguishing between its various presentations.

### 3.3. Treatment

Treatment can be planned depending upon the presentation, localization, and size of the tumor. There is a significantly lower rate of progression to leukemia and longer survival among patients who received any form of chemotherapy at diagnosis of GS [17]. There is a general consensus that all these patients must receive standard systemic induction-intensification chemotherapy regimens similar to those given in acute myeloid leukemia [5, 7, 18]. Surgical excision and radiotherapy (tumor is highly radiosensitive) can be curative, but mostly are performed to reduce the bulk the of tumor and to relieve the compressive symptoms. High dose methylprednisolone treatment has been reported to markedly reduce the size of the tumor. Other adjuvant treatment modalities that have been applied successfully include low dose alpha-interferon and disodium pamidronate, low dose methotrexate, allogeneic bone marrow transplant, and autologous stem cell transplantation [5, 18].

Reports have emphasized the use of combined local and systemic radical options, including chemotherapy, radiation therapy, surgical intervention, and bone marrow transplantation. Because of information on a limited number of patients, there is no agreed upon optimal therapy. However, systemic chemotherapy similar to that given for acute myeloid leukemia with or without local radiotherapy may result in long remissions [18, 19].

### 3.4. Prognosis

Generally, GS is associated with decreased overall survival. Blastic types, age more than 50 years, and extramedullary relapse following allogeneic bone marrow transplant are significant adverse prognostic factors [5, 12, 19]. The median overall survival is about 20–22 months [12, 13]. The median survival of patients with chromosome 8 abnormalities is 12 months [13]. In our patient, chromosome analysis revealed complex clonal aberrations with deletion of 5q. Deletion of 5q occurs in MDS/AML. In the International Scoring System, MDS patients with this karyotype pattern were placed in a poor prognostic category [20].

## 4. Conclusion

GS is difficult to recognize and may be easily overlooked or misdiagnosed. GS may precede the diagnosis of a chronic myeloproliferative disorder or acute myeloid leukemia, or may present parallel to a hematologic diagnosis. An accurate diagnosis of GS is of great clinical importance in the ongoing management of hematologic malignancies. Precise diagnosis is essential because all GS should be treated as acute myeloid leukemias. Immunohistochemical and enzyme histochemical staining are useful in establishing the diagnosis, although suspicion of the diagnosis on examination of routinely stained sections is of paramount importance.

This case highlights a rare hematological cancer that a clinician should consider when a patient presents with a breast mass. Our objective of presenting this case is to enhance awareness of GS in personnel providing health care. Increased clinical awareness of this entity will facilitate early diagnosis. A high index of suspicion is required and timely recognition of GS is important, because aggressive induction chemotherapy or radiation therapy can affect outcome, minimizing potentially preventable patient morbidity and mortality.
